# Baicalin and Baicalein Enhance Cytotoxicity, Proapoptotic Activity, and Genotoxicity of Doxorubicin and Docetaxel in MCF-7 Breast Cancer Cells

**DOI:** 10.3390/molecules29112503

**Published:** 2024-05-25

**Authors:** Joanna Bernasinska-Slomczewska, Pawel Hikisz, Anna Pieniazek, Aneta Koceva-Chyla

**Affiliations:** 1 Department of Oncobiology and Epigenetics, Faculty of Biology and Environmental Protection, University of Lodz, Pomorska 141/143 Str., 90-236 Lodz, Poland; pawel.hikisz@biol.uni.lodz.pl (P.H.); anna.pieniazek@biol.uni.lodz.pl (A.P.); 2 Department of Medical Biophysics, Faculty of Biology and Environmental Protection, University of Lodz, Pomorska 141/143 Str., 90-236 Lodz, Poland; koceva.aneta@gmail.com

**Keywords:** baicalin, baicalein, doxorubicin, docetaxel, cytotoxicity, apoptosis, DNA damage

## Abstract

Breast cancer is a major health concern and the leading cause of death among women worldwide. Standard treatment often involves surgery, radiotherapy, and chemotherapy, but these come with side effects and limitations. Researchers are exploring natural compounds like baicalin and baicalein, derived from the *Scutellaria baicalensis* plant, as potential complementary therapies. This study investigated the effects of baicalin and baicalein on the cytotoxic, proapoptotic, and genotoxic activity of doxorubicin and docetaxel, commonly used chemotherapeutic drugs for breast cancer. The analysis included breast cancer cells (MCF-7) and human endothelial cells (HUVEC-ST), to assess potential effects on healthy tissues. We have found that baicalin and baicalein demonstrated cytotoxicity towards both cell lines, with more potent effects observed in baicalein. Both flavonoids, baicalin (167 µmol/L) and baicalein (95 µmol/L), synergistically enhanced the cytotoxic, proapoptotic, and genotoxic activity of doxorubicin and docetaxel in breast cancer cells. In comparison, their effects on endothelial cells were mixed and depended on concentration and time. The results suggest that baicalin and baicalein might be promising complementary agents to improve the efficacy of doxorubicin and docetaxel anticancer activity. However, further research is needed to validate their safety and efficacy in clinical trials.

## 1. Introduction

Nowadays, cancer is considered the leading cause of death worldwide, partly due to marked declines in the mortality rates of stroke and coronary heart disease relative to cancer in many countries. The World Health Organization (WHO) estimated 10 million cancer deaths (nearly one in six deaths) in 2020. Breast cancer was the most frequently diagnosed cancer in women in 2020, with an estimated 2.3 million new cases (11.7% of total cases), and the fifth leading cause of cancer mortality worldwide, with 685,000 deaths (6.9% of total cases) [1]. The popularization of early detection programs for breast cancer, the increased awareness of symptoms, and the development of different modes of treatment have proven successful in high-income countries where the five-year survival rate is 91–99% if the disease does not spread to lymph nodes [2].

Breast cancer treatment is determined by the condition of patients; disease stage; and clinical and molecular characteristics of the tumor and involves either breast-conserving surgery or mastectomy; radiotherapy; and systemic therapy. Systemic therapy refers to cancer treatments that vary in their mechanisms of action and include chemotherapy; endocrine (hormone) therapy; targeted biologic therapy (antibodies); and, more recently, immunotherapy [3]. These medications are utilized in adjuvant, neoadjuvant, and metastatic settings. Generally, systemic agents exhibit efficacy at the onset of therapy in 90% of primary breast cancers and 50% of metastases. Nonetheless, approximately 30% of patients with early-stage breast cancer experience recurrent disease [4].

Chemotherapy remains the gold standard for treating breast cancer; anthracycline-based regimens are preferred, and the addition of taxanes increases the response rate and survival rate in patients with metastatic cancer. Anthracyclines are compounds originally derived from *Streptomyces*, and their antitumor activities were established in 1960 and were named doxorubicin and daunorubicin [5,6]. The mechanisms by which anthracyclines inhibit the growth of cancer cells involve multiple pathways, such as (1) intercalation into DNA, which leads to the inhibition of the synthesis of macromolecules; (2) the generation of free radicals that cause DNA damage or lipid peroxidation; (3) DNA binding and alkylation; (4) DNA cross-linking; (5) interference with DNA unwinding or DNA strand separation and helicase activity; (6) the direct effects on cell membranes; (7) the initiation of DNA damage via inhibition of topoisomerase II; and (8) the induction of apoptosis in response to topoisomerase II inhibition [7]. However, the clinical utility of anthracyclines is limited by cumulative dose-dependent cardiac toxicity mediated by reactive oxygen species (ROS), often resulting in lethal congestive heart failure. Taxanes are microtubule inhibitors that can induce apoptosis and inhibit tumor angiogenesis. Paclitaxel, extracted from the Pacific yew (*Taxus brevifolia*) in 1971, and docetaxel, a semi-synthetic taxane analog developed from the European yew (*Taxus baccata*) in 1980, are two prominent examples of taxanes [8,9]. Docetaxel binds with greater affinity to β-tubulin and exhibits a broader range of activity than paclitaxel. Furthermore, due to its greater uptake and slower efflux, it has a longer retention time in tumor cells than paclitaxel and promotes apoptotic cell death via the phosphorylation of Bcl-2 [10].

Although anthracyclines and taxanes are effective cytostatics and play an established role in breast cancer treatment; their use is often limited by the development of resistance in tumor cells; side effects; and high systemic toxicity. This confines the total tolerated drug dose, negatively influencing patient outcomes. As a result, the employment of these chemotherapeutics is a balance between absolute benefits and risks. The heterogeneity of risk factors has made breast cancer treatment challenging, particularly in patients with advanced and metastatic stages [11,12]. Due to these facts, developing new strategies and therapeutic regimens in adjuvant therapy is needed. In an effort to develop such effective strategies, some patients are being directed toward phytotherapy, mainly because of the association of phytotherapeutic agents with a wide range of health-promoting effects [13]. Many naturally occurring agents have shown not only chemopreventive and chemotherapeutic but also antioxidant and anti-inflammatory potential in various bioassay systems and animal models. These beneficial effects are often linked with their potentially non-toxic effect in normal cells, capability of oral consumption, low cost, and acceptance by the human population [14,15].

*Scutellaria baicalensis Georgi* is a flowering plant in the *Lamiaceae* family, which mainly grows in China, Russia’s Eastern Siberia, Mongolia, North Korea, and Japan. The dried roots of this medicinal plant, called Scutellariae radix, are a well-known herbal drug of East Asian phytotherapy systems used to treat inflammation, hypertension, cardiovascular diseases, and bacterial and viral infections [16,17]. *Scutellaria baicalensis Georgi* contains various natural products, including flavonoids, diterpenes, polyphenols, amino acids, essential oils, sterols, and benzoic acids [18]. Among nearly 60 flavonoid structures identified in *Scutellaria baicalensis*, baicalein and wogonin, along with their glucuronides (baicalin and wogonoside), are the major bioactive compounds [19].

Baicalin and baicalein have attracted great attention in the cosmetic, food, and pharmaceutical industries because of the wide range of their beneficial effects and clinical applications. Several studies have shown that flavonoids such as baicalin and baicalein have many beneficial pharmacological properties. These properties include anti-viral, anti-bacterial, antioxidative, anticonvulsant, hepatoprotective, and neuroprotective effects [20]. Baicalin and baicalein can scavenge reactive oxygen species (ROS) and interact with various signaling molecules involved in processes such as apoptosis, inflammation, autophagy, cell cycle, mitochondrial dynamics, and cytoprotection [16,21]. Additionally, baicalin and baicalein exhibit promising antitumor activity, both in vitro in cancer cell lines and in vivo in animal models, by multiple routes of therapeutic action. These therapeutic actions include inducing apoptosis and tumor cell cycle arrest, inhibiting tumor migration and invasion, suppressing tumor angiogenesis, and overcoming drug resistance [16,22].

It is supposed that the co-administration of phytotherapeutic agents possessing a wide range of pharmacological activities with commonly used anticancer drugs might be a new approach for effective cancer therapies or adjuvant treatments without compromising therapeutic efficacy but improving patient survival and quality of life. Thus, the aim of this study was the evaluation of the influence of baicalin or its aglycone baicalein on the cytotoxic, proapoptotic, and genotoxic activity of anticancer drugs such as anthracyclines (doxorubicin) or taxanes (paclitaxel) in the breast cancer cell line (MCF-7) and human endothelial cell line HUVEC-ST. On the other hand, in vitro studies concerning the effects of such complementary treatment based on doxorubicin or taxanes in combination with baicalin or baicalein are crucial because the use of different types of complementary therapies has been increasingly popular among breast cancer patients [23]. Last but not least, the immortalized human umbilical vein endothelial cells (HUVEC-ST) primarily isolated from the vein of the umbilical cord were used in this study to assess the potential effects of the treatment based on flavonoid-anticancer drug combinations on the endothelium and to identify any possible safety concerns, which serves as a novelty. The endothelial cells are a type of cells that is sensitive to the effects of chemotherapy drugs and might be damaged during the intravenous application of chemotherapeutics. The dysfunction of endothelial cells may lead to inflammation, blood clots, and other problems.

## 2. Results

### 2.1. Cytotoxicity of Investigated Compounds

#### 2.1.1. Baicalin and Baicalein

**Figure 1:**
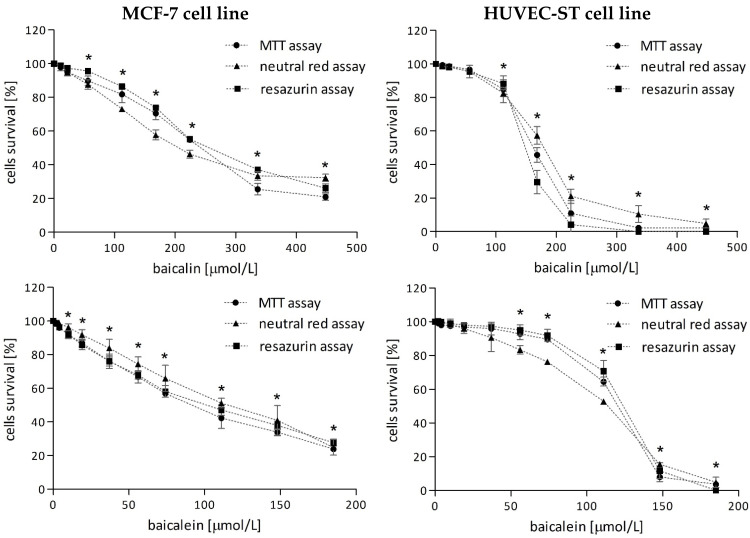
Survival curves of MCF-7 (**left panel**) and HUVEC-ST (**right panel**) cells after 24h-incubation with increasing concentrations of baicalin and baicalein. Cell viability was measured using MTT, resazurin, and neutral red method. Data are shown as means ± SD from four individual experiments for MCF-7 and HUVEC-ST cells, with each repeated at least six times. * *p* < 0.05 vs. control.

In both investigated cell lines, breast cancer and endothelial, 24-h incubation with increasing concentrations of baicalin (from 11 µmol/L to 448 µmol/L) or baicalein (from 2 µmol/L to 185 µmol/L) caused a progressive reduction of the number of living cells (Figure 1). However, the cytotoxicity of baicalein was more powerful. This effect was observed independently from the applied method: MTT assay, neutral red uptake assay, or resazurin reduction assay.

Surprisingly, low concentrations of baicalin and baicalein had a greater effect on MCF-7 viability. A statistically significant reduction in the number of living breast cancer cells was observed after treatment with baicalin at concentrations ≥ 56 µmol/L and baicalein at concentrations ≥ 10 µmol/L. Meanwhile, baicalin at concentrations ≥ 112 µmol/L and baicalein at concentrations ≥ 56 µmol/L significantly decreased HUVEC-ST viability. This effect can be observed as a long shoulder (especially for baicalein) on the survival curves of endothelial cells.

Based on the survival curves obtained by the MTT method, the baicalin and baicalein inhibitory concentrations IC_10_ (a concentration of a compound that reduces cell viability by 10%) and IC_50_ (a concentration of a compound that reduces cell viability by 50%) were calculated (Table 1). Due to the diverse cytotoxicity of flavonoids towards investigated cell lines, the subsequent concentrations of baicalin IC_10_ = 36 µmol/L, IC_50_ = 167 µmol/L and baicalein IC_10_ = 9 µmol/L, IC_50_ = 95 µmol/L were chosen for further experiments.

#### 2.1.2. Anticancer Drugs

**Figure 2:**
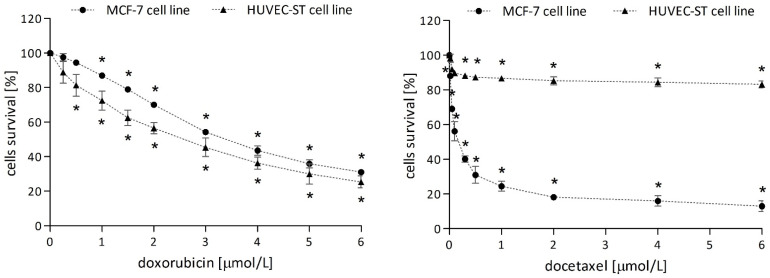
Survival curves of MCF-7 and HUVEC-ST cells after 2-h incubation with increasing concentrations of doxorubicin or docetaxel. Cell viability was measured by MTT assay. Values are presented as means ± SD from four individual experiments for MCF-7 and HUVEC-ST cells, with each repeated at least six times. * *p* < 0.05 vs. control.

Doxorubicin showed similar cytotoxicity towards MCF-7 and HUVEC-ST cells. The drug caused a statistically significant decrease in the survival of cancer cells at concentrations ≥ 1 µmol/L and of endothelial cells at concentrations ≥ 0.5 µmol/L (Figure 2, left panel).

Docetaxel showed more significant cytotoxicity towards breast cancer cells, causing a statistically significant decrease in survival at concentrations ≥ 0.02 µmol/L (Figure 2, right panel). The highest taxane concentration (6 µmol/L) was caused only a 20% decrease in the survival of human endothelial cells. In further experiments, both cell lines were treated with drug IC_50_ concentration determined based on survival curves obtained by the MTT method: doxorubicin = 3 µmol/L and docetaxel = 0.1 µmol/L.

#### 2.1.3. The Effect of Baicalin and Baicalein on the Cytotoxicity of Anticancer Drugs

**Figure 3:**
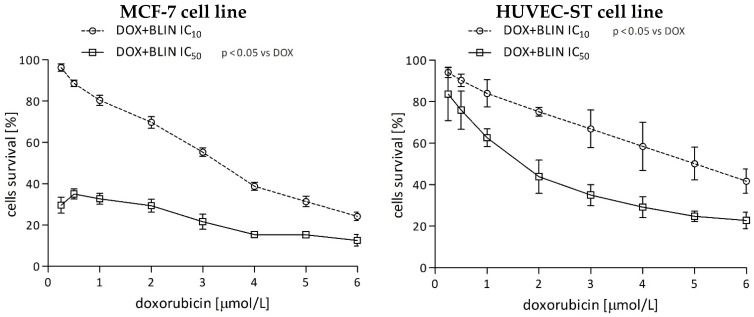

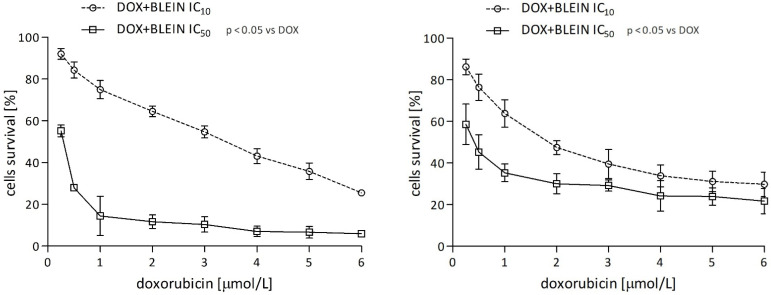
Survival curves of MCF-7 (**left panel**) and HUVEC-ST (**right panel**) cells preincubated for 22 h with IC_10_ and IC_50_ concentrations of baicalin or baicalein and then incubated for 2 h with DOX at concentrations 0.25, 0.5, 1, 2, 3, 4, 5, and 6 μmol/L. Cell viability was measured by MTT assay. Values are presented as means ± SD from four individual experiments for MCF-7 and HUVEC-ST cells, with each repeated at least six times. Statistical significance in the legend indicates that the preincubation with IC_10_ and IC_50_ concentrations of baicalin or baicalein shifts the basal survival DOX curve.

The 22-h preincubation with high concentrations of baicalin or baicalein (IC_50_) strongly enhances doxorubicin anticancer activity towards breast cancer cells (Figure 3, left panel). The interaction between the drug and flavonoids had a synergistic character, and the calculated CI index was 0.64347 for DOX IC_50_ + BLIN IC_50_ and 0.34117 for DOX IC_50_ + BLEIN IC_50_ combinations (Table 2). A low concentration of baicalin or baicalein (IC_10_) did not affect anthracycline toxicity in MCF-7 cells. A robust potentiation of drug cytotoxicity by flavonoids was not observed in endothelial cells (Figure 3, right panel). Regarding HUVEC-ST cells, the CI index calculated for the combination of DOX IC_50_ + BLIN IC_50_ was 1.79361, indicating antagonist interaction, and for the combination of DOX IC_50_ + BLEIN IC_50_, it was 0.68010, indicating synergistic relation (Table 2). Additionally, a low concentration (IC_10_) of baicalin significantly diminished doxorubicin toxicity. The CI index value for this combination was 2.91523, which strongly signifies antagonist relation. Such an effect was not observed in the case of a low concentration (IC_10_) of baicalein.

**Figure 4:**
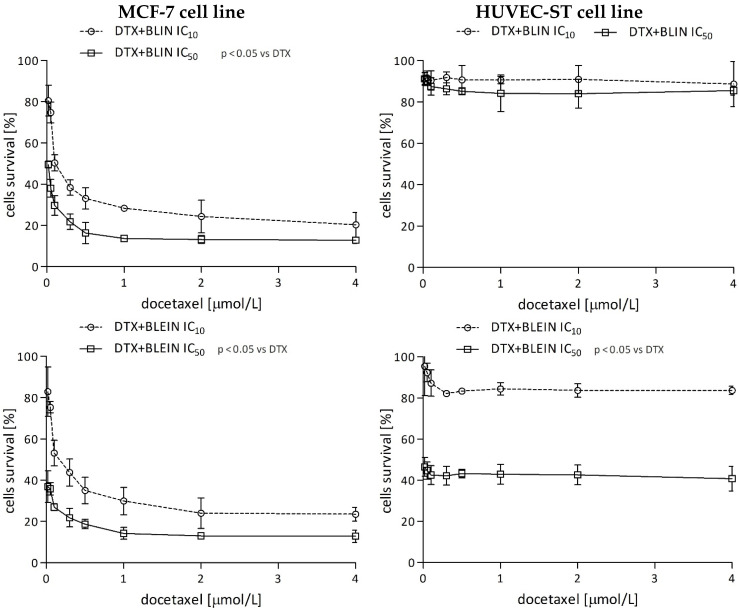
Survival curves of MCF-7 (**left panel**) and HUVEC-ST (**right panel**) cells preincubated for 22 h with IC_10_ and IC_50_ concentrations of baicalin or baicalein and then incubated for 2 h with DTX at concentrations 0.02, 0.05, 0.1, 0.3, 0.5, 1, 2, 4 μmol/L. Cell viability was measured by MTT assay. Values are presented as means ± SD from four individual experiments for MCF-7 and HUVEC-ST cells, with each repeated at least six times. Statistical significance in the legend indicates that the preincubation with IC_10_ and IC_50_ concentrations of baicalin or baicalein shifts the basal survival DTX curve.

Similar to effects observed during the treatment of MCF-7 cells with a combination of doxorubicin and flavonoids, high concentrations of baicalin and baicalein also potentiate taxane anticancer activity (Figure 4, left panel). The interaction between the taxane and flavonoids had a synergistic character, and the calculated CI index was 0.54206 for DTX IC_50_ + BLIN IC_50_ and 0.62760 for DTX IC_50_ + BLEIN IC_50_ combinations (Table 2). A low concentration of baicalin or baicalein (IC_10_) did not affect taxane toxicity in breast cancer and endothelial cells. It is noteworthy that the IC_50_ concentration of baicalin did not affect docetaxel toxicity in HUVEC-ST cells (Figure 4, right panel). The CI index value for this combination was 3.13654, which strongly signifies antagonist relation (Table 2). In contrast, the IC_50_ concentration of baicalein enhanced docetaxel toxicity towards HUVEC-ST cells, and the calculated CI index was 0.44278, suggesting synergism.

### 2.2. The Influence of Baicalin and Baicalein on Doxorubicin Accumulation

**Figure 5:**
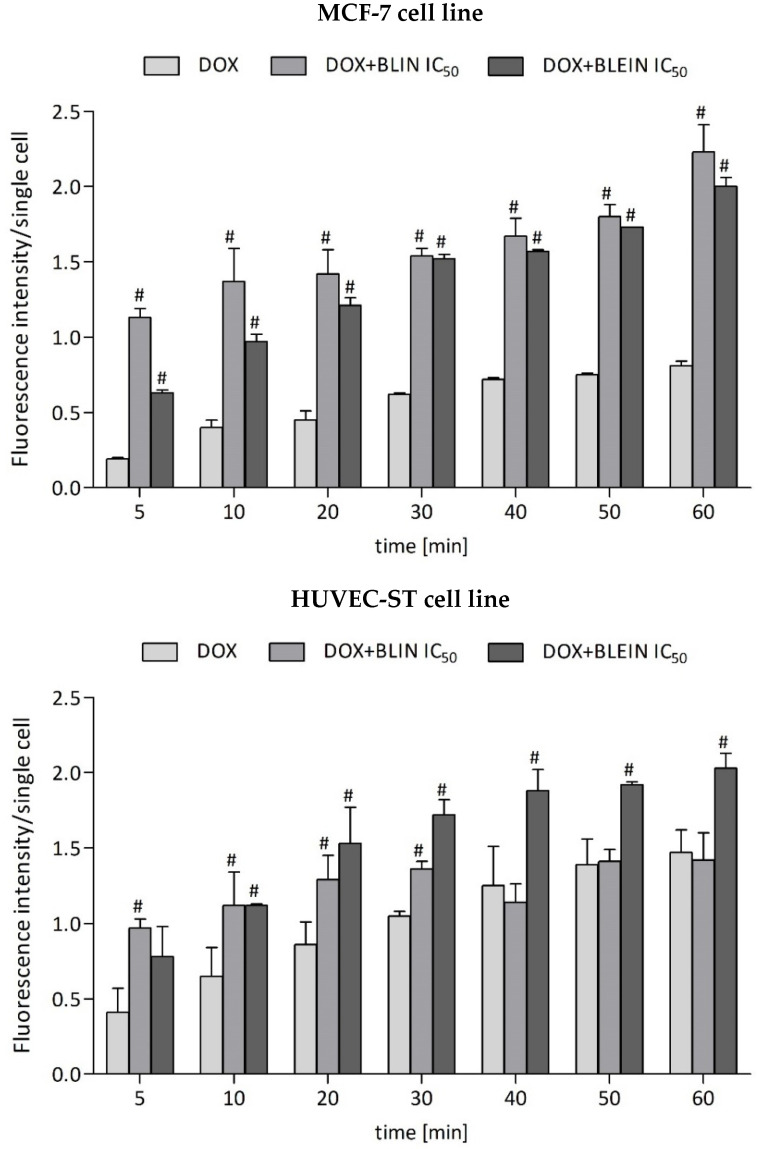
Doxorubicin accumulation in MCF-7 and HUVEC-ST cells after 22 h of incubation with IC_50_ concentration of BLIN or BLEIN. Values are presented as means ± SD from four individual experiments for MCF-7 and HUVEC-ST cells. Doxorubicin fluorescence intensity was calculated per single cell. # *p* < 0.05 vs. DOX.

Both investigated flavonoids significantly increased doxorubicin influx into breast cancer cells (Figure 5). After 20 min of drug management, the amount of doxorubicin was over two times higher in cells preincubated for 22 h with IC_50_ concentrations of baicalin or baicalein relative to cells not treated with flavonoids. For the first 20 min, baicalin had a higher impact on drug accumulation. However, after that time, the enhancement of doxorubicin accumulation inside MCF-7 was comparable between cells preincubated with baicalin or baicalein. According to endothelial cells, increased doxorubicin accumulation was maintained for the whole study period after preincubation for 22 h with an IC_50_ concentration of baicalein (Figure 5). In the case of baicalin, a significant enhancement of drug influx into HUVEC-ST cells was observed only during the first 30 min of the study.

### 2.3. Induction of Apoptosis and Necrosis by Baicalin and Baicalein Used alone and in Combination with Anticancer Drugs

#### 2.3.1. MCF-7 Cell Line

**Figure 6:**
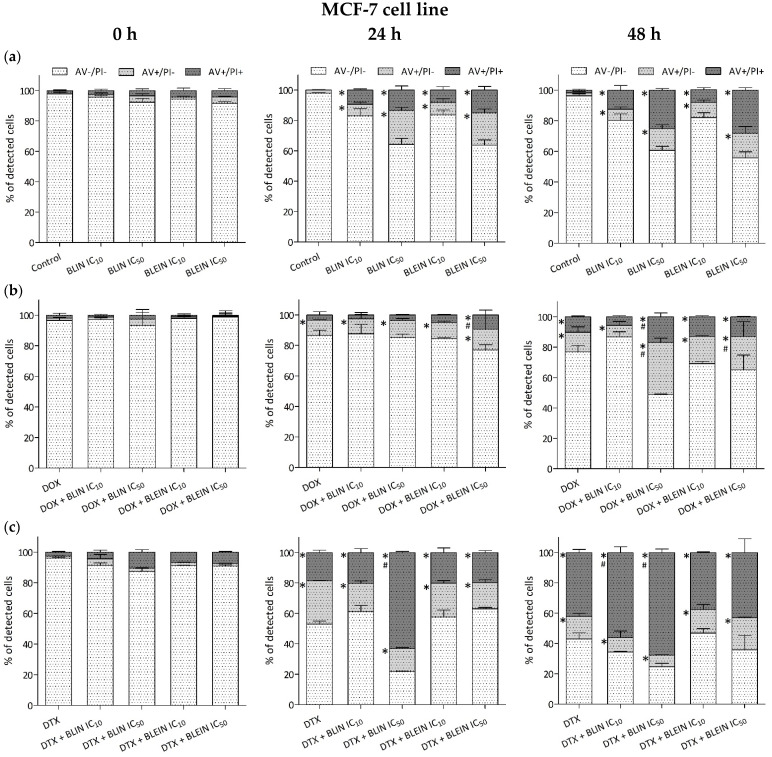
Changes in membrane lipid asymmetry related to induction of apoptosis and necrosis in MCF-7 cells after (**a**) 24 h incubation with IC_10_ or IC_50_ concentration of BLIN or BLEIN, (**b**) 2 h of incubation with IC_50_ concentrations of DOX alone or after 22 h of preincubation with IC_10_ or IC_50_ concentration of BLIN or BLEIN, and (**c**) 2 h of incubation with IC_50_ concentrations of DTX alone or after 22 h of preincubation with IC_10_ or IC_50_ concentration of BLIN or BLEIN. The measurements were conducted immediately after incubation and 24 or 48 h later. Values are presented as means ± SD from four individual experiments. * *p* < 0.05 vs. control, # *p* < 0.05 vs. drug used alone.

Baicalin and baicalein caused significant changes in membrane lipid asymmetry related to induction apoptosis and necrosis in the MCF-7 cells in a concentration-dependent manner (Figure 6a). Statistically significant changes were observed 24 and 48 h after the incubation ended. The highest percentage of AV+/PI− cells, which undergo apoptosis (average 20%), was observed 24 h after incubation with an IC_50_ concentration of baicalin or baicalein. However, 48 h after incubation, the AV+/PI+ cells were in the dominant part (BLIN IC_50_—25%, BLEIN IC_50_—28%). The fraction of AV+/PI− or AV+/PI+ cells in samples exposed to lower concentrations (IC_10_) of flavonoids was close to 10% in all of the analyzed post-incubation times.

Doxorubicin alone or in combination with flavonoids caused a decrease in the survival of MCF-7 cells, mainly by the induction of apoptosis (Figure 6b). Statistically significant changes in the number of AV+/PI− or AV+/PI+ cells were observed after 24 and 48 h of post-incubation culture. The enhancement of anthracycline proapoptotic activity was observed during the longest post-incubation time (48 h). The percentage of AV+/PI− cells that undergo apoptosis was 34% for the drug combination with baicalin at IC_50_ concentration and 21% for the drug combination with baicalein at IC_50_ concentration. In contrast, doxorubicin alone affected 12% of the cell population by inducing apoptosis. Lower concentrations (IC_10_) of flavonoids did not influence the anthracycline proapoptotic activity.

After treatment with docetaxel in combination with baicalin or baicalein, a statistically significant decrease in the amount of viable MCF-7 cells and an increase in the number of AV+/PI− or AV+/PI+ cells were observed after 24 and 48 h of post-incubation culture (Figure 6c). Only baicalin enhanced taxane cytotoxic activity. In cells treated with docetaxel combined with a high concentration of baicalin, the percentage of viable cells decreased from 53% to 22% (24 h after the incubation) and from 43% to 24% (48 h after the incubation). The fraction of cells that exhibited significant changes in membrane lipid asymmetry—Annexin-V-positive—mainly consisted of AV+/PI+ cells.

#### 2.3.2. HUVEC-ST Cell Line

**Figure 7:**
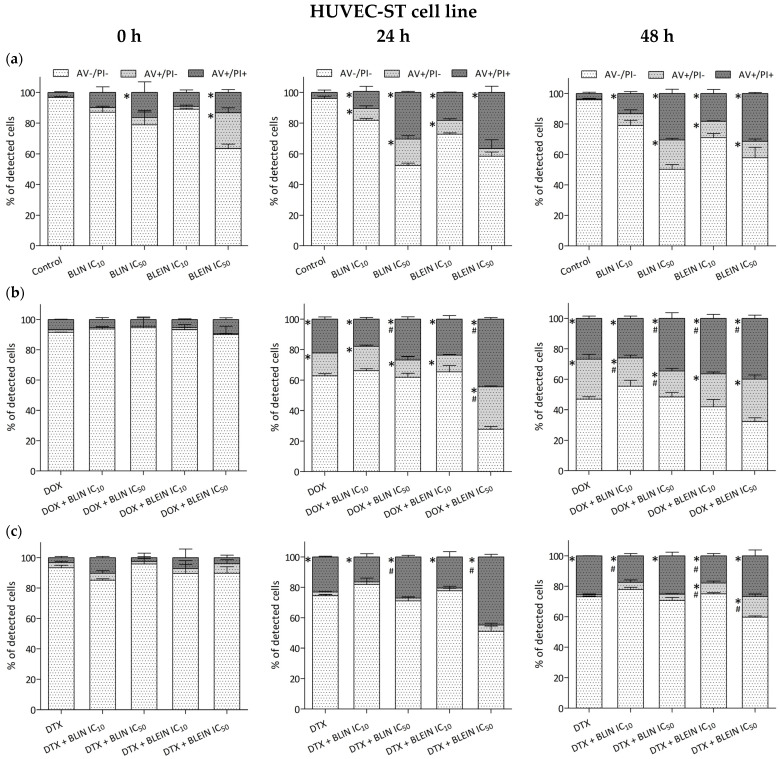
Changes in membrane lipid asymmetry related to induction of apoptosis and necrosis in HUVEC-ST cells after (**a**) 24 h incubation with IC_10_ or IC_50_ concentration of BLIN or BLEIN, (**b**) 2 h of incubation with IC_50_ concentrations of DOX alone or after 22 h of preincubation with IC_10_ or IC_50_ concentration of BLIN or BLEIN, and (**c**) 2 h of incubation with IC_50_ concentrations of DTX alone or after 22 h of preincubation with IC_10_ or IC_50_ concentration of BLIN or BLEIN. The measurements were conducted immediately after incubation and 24 or 48 h later. Values are presented as means ± SD from four individual experiments. * *p* < 0.05 vs. control, # *p* < 0.05 vs. drug used alone.

Similarly to cancer cells, baicalin and baicalein caused significant changes in membrane lipid asymmetry related to induction apoptosis and necrosis in the HUVES-ST cells in a concentration-dependent manner (Figure 7a). Statistically significant changes were observed in all of the investigated postincubation times, mainly after treatment with high concentrations of flavonoids. Forty-eight hours after incubation with flavonoids, the fraction of Annexin-V-positive cells in the samples was AV+/PI−—19%, AV+/PI+—30% for baicalin and AV+/PI−—10%, AV+/PI+ –31% for baicalein.

After the treatment of endothelial cells with doxorubicin alone or in combination with flavonoids, statistically significant changes in the number of AV+/PI− or AV+/PI+ cells were observed after 24 and 48 h of post-incubation culture (Figure 7b). During the mentioned postincubation times, the most vital enhancement of anthracycline cytotoxic activity was observed after incubation with doxorubicin in combination with baicalein at IC_50_ concentration. Twenty-four hours after the incubation, the percentage of AV+/PI− or AV+/PI+ increased almost two-fold. In contrast, during the longest postintubation time, in samples treated with a combination of anticancer drug and baicalin, a slight decrease in the amount of AV+/PI− with a simultaneous increase in the percentage of viable cells was observed. In cells preincubated with a lower concentration of baicalin, the percentage of viable cells increased from 47% to 55% compared to doxorubicin used alone.

Docetaxel alone or combined with flavonoids caused changes in the membrane lipid asymmetry of HUVEC-ST cells. Statistically significant changes in the number of AV+/PI+ cells were observed in all investigated samples after 24 and 48 h of post-incubation culture (Figure 7c). Baicalin and baicalein at high concentrations enhanced the cytotoxic activity of taxane. Twenty-four hours after the incubation, in cells treated with docetaxel combined with baicalein at IC_50_ concentration, the AV+/PI+ cells increased from 23% to 44%. In contrast, after the longest postincubation time in HUVEC-ST cells treated with a combination of docetaxel and flavonoids in low concentration, there was a slight decrease in AV+/PI+ cells from 26% to 18%. A statistically significant increase in the number of AV+/PI− cells was observed only 48 h after the treatment of endothelial cells with taxane in combination with baicalein.

### 2.4. Changes in Mitochondrial Membrane Potential Caused by Baicalin and Baicalein Used alone and in Combination with Anticancer Drugs

**Figure 8:**
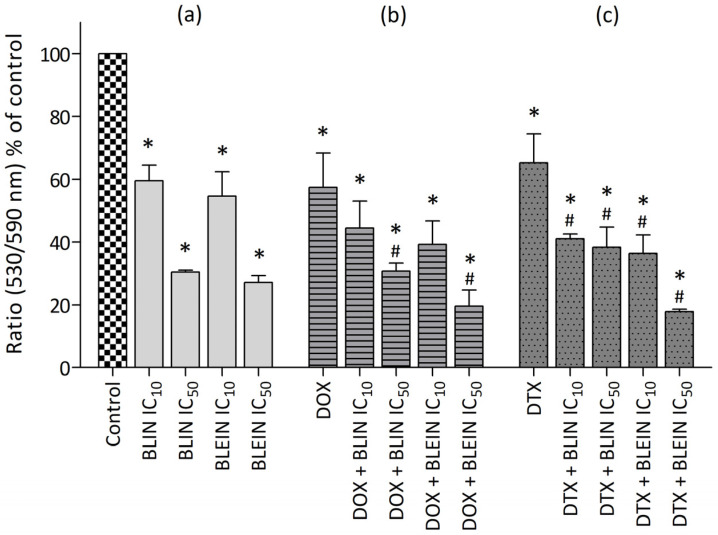
Changes in mitochondrial membrane potential in MCF-7 cells after (**a**) 24 h incubation with IC_10_ or IC_50_ concentration of BLIN or BLEIN, (**b**) 2 h of incubation with IC_50_ concentrations of DOX alone or after 22 h of preincubation with IC_10_ or IC_50_ concentration of BLIN or BLEIN, and (**c**) 2 h of incubation with IC_50_ concentrations of DTX alone or after 22 h of preincubation with IC_10_ or IC_50_ concentration of BLIN or BLEIN. Data are presented as means ± SD from four individual experiments, with each repeated at least six times. Control cells were arbitrarily taken as 100%. * *p* < 0.05 vs. control, # *p* < 0.05 vs. drug used alone.

**Figure 9:**
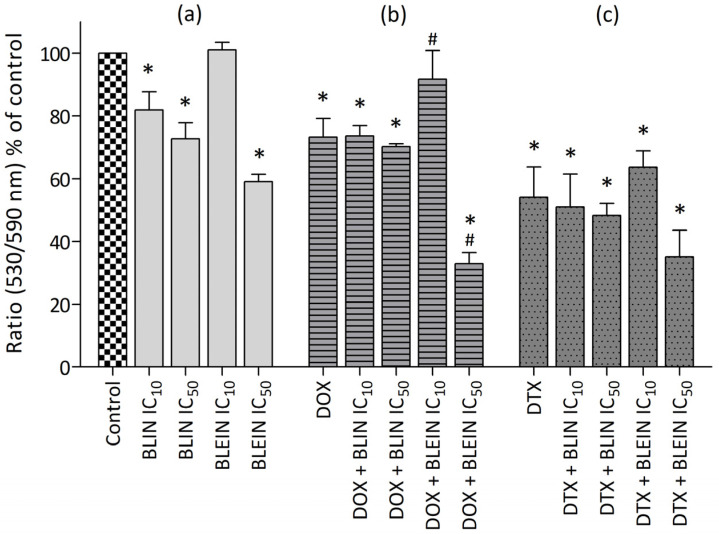
Changes in mitochondrial membrane potential in HUVEC-ST cells after (**a**) 24 h incubation with IC_10_ or IC_50_ concentration of BLIN or BLEIN, (**b**) 2 h of incubation with IC_50_ concentrations of DOX alone or after 22 h of preincubation with IC_10_ or IC_50_ concentration of BLIN or BLEIN, and (**c**) 2 h of incubation with IC_50_ concentrations of DTX alone or after 22 h of preincubation with IC_10_ or IC_50_ concentration of BLIN or BLEIN. Data are presented as means ± SD from four individual experiments, with each repeated at least six times. Control cells were arbitrarily taken as 100%. * *p* < 0.05 vs. control, # *p* < 0.05 vs. drug used alone.

Both investigated flavonoids significantly decreased the mitochondrial membrane potential of MCF-7 cells in a concentration-dependent manner. After treatment with baicalin and baicalein at high concentrations (IC_50_), the ΔΨm of cancer cells was diminished to 30% of the control value (Figure 8a). In contrast, the ΔΨm of HUVEC-ST cells treated with baicalin and baicalein at high concentrations (IC_50_) did not undergo such a substantial decrease. The measured mitochondrial membrane potential in endothelial cells incubated with baicalin and baicalein in IC_50_ concentration was reduced to 72% and 59%, respectively (Figure 9a).

Doxorubicin significantly influenced the mitochondrial membrane potential of MCF-7 cells and HUVEC-ST cells. After treatment with anthracycline, ΔΨm was reduced to 57% in cancer cells and 73% in endothelial cells compared to the value of control cells (100%). In MCF-7 cells, preincubation with high concentrations of baicalin and baicalein significantly enhanced doxorubicin activity, and the ΔΨm was reduced to 31% and 20%, respectively. According to HUVEC-ST cells, the parameter was significantly diminished to 33% after treatment with anthracycline combined with baicalein in concentration IC_50_. Surprisingly, preincubation with baicalein in low concentration exhibited the opposite effect on HUVEC-ST cells by increasing ΔΨm to the level of control (Figure 8b and Figure 9b).

A significant decrease in mitochondrial membrane potential was observed after treating breast cancer and endothelial cells with docetaxel. The ΔΨm parameter was reduced to 65% in MCF-7 cells and 54% in HUVEC-ST. The preincubation of cancer cells with baicalin or baicalin significantly enhanced taxane activity. The highest reduction of mitochondrial membrane potential, ΔΨm = 18%, was observed after treatment with docetaxel combined with baicalein in IC_50_ concentration. According to the HUVEC-ST cells, none of the investigated flavonoids influenced changes in mitochondrial membrane potential caused by taxane (Figure 8c and Figure 9c).

### 2.5. DNA Damage Induced by Baicalin and Baicalein Used Alone and in Combination with Anticancer Drugs

**Figure 10:**
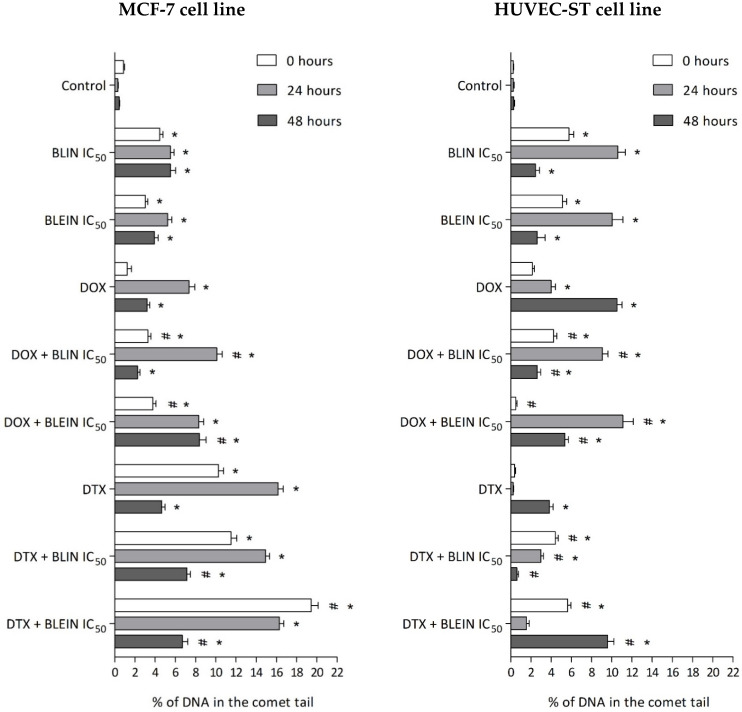
The percent of DNA in the comet tail of MCF-7 (**left panel**) and HUVEC-ST (**right panel**) cells after 24 h incubation with IC_50_ concentration of BLIN or BLEIN, 22 h incubation with IC_50_ concentration of BLIN or BLEIN and subsequently 2 h with IC_50_ concentrations of DOX, or 22 h incubation with IC_50_ concentration of BLIN or BLEIN and subsequently 2 h with IC_50_ concentrations of DTX. The measurements were conducted immediately after incubation and 24 or 48 h later. Data are presented as means ± SD from four individual experiments. * *p* < 0.05 vs. control, # *p* < 0.05 vs. drug used alone.

As shown in Figure 10, high concentrations (IC_50_) of baicalin and baicalein used alone and in combination with anticancer drugs exhibited genotoxic potential by the induction of statistically significant damages in the DNA of MCF-7 and HUVEC-ST cells.

Anticancer drugs used alone exhibited diverse genotoxicity. Immediately after incubation with doxorubicin, DNA damage was not observed in MCF-7 and HUVEC-ST cells. During longer postincubation times, doxorubicin-induced DNA breaks in both cell types (Table 3, Photo a). The highest percentage—10% of DNA in the comet tail—was observed in endothelial cells 48 h after incubation (Table 3, Photo b). In contrast, docetaxel genotoxic properties were mainly observed in MCF-7 cells. The highest amount of DNA in the comet tail was observed in MCF-7 cells immediately and 24 h after incubation with taxane (Table 3, Photo c). Simultaneously, during the mentioned postincubation times, the anticancer drug did not cause statistically significant damage to the DNA of endothelial cells (Table 3, Photo d). Only the combination of taxane with baicalein (IC_50_) strongly enhances taxane genotoxicity, especially 48 h after incubation.

During all of the investigated postincubation times, the percentage of DNA breaks in MCF-7 cells treated with flavonoids did not exceed 6% (Table 3, Photo e). The damage level observed in endothelial cells 24 h after incubation was twice as high (Table 3, Photo f).

According to the MCF-7 cells, the highest level of DNA damage was observed immediately, and 24 h after incubation with docetaxel and baicalein (IC_50_), the percentage of DNA in the comet tail was 19% and 16%, respectively (Table 3, Photo g). The highest level of DNA in the comet tail, 19%, resulted from the significant enhancement of taxane genotoxicity by baicalein. Both flavonoids enhance damages caused by drugs alone; however, the potentiation was diverse between investigated postincubation times. Interestingly, a significant reduction of DNA damage was observed during the longest postincubation time after the treatment of HUVEC-ST cells with doxorubicin combined with baicalin or baicalein. The amount of DNA in the comet tail decreased from 10% to 3% after preincubation with baicalin and from 10% to 5% after preincubation with baicalein, compared to the drug used alone (Table 3, Photo i).

### 2.6. PARP Cleavage Induced by Baicalin and Baicalein Used Alone and in Combination with Anticancer Drugs

**Figure 11:**
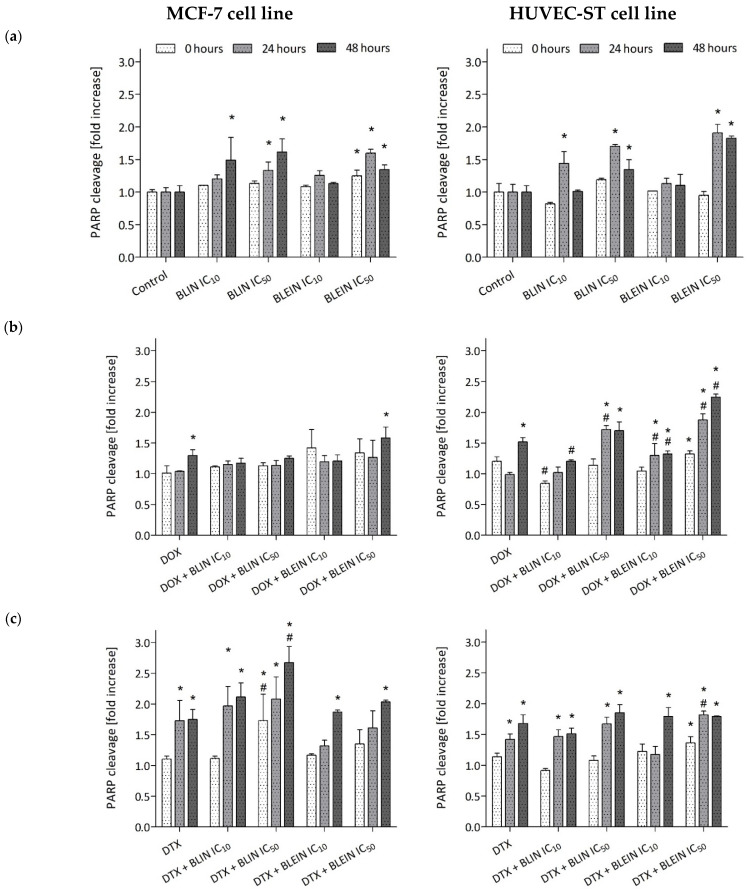
PARP cleavage quantification in MCF-7 (**left panel**) and HUVEC-ST (**right panel**) cells after (**a**) 24 h incubation with IC_10_ or IC_50_ concentration of BLIN or BLEIN, (**b**) 2 h of incubation with IC_50_ concentrations of DOX alone or after 22 h of preincubation with IC_10_ or IC_50_ concentration of BLIN or BLEIN, and (**c**) 2 h of incubation with IC_50_ concentrations of DTX alone or after 22 h of preincubation with IC_10_ or IC_50_ concentration of BLIN or BLEIN. The measurements were conducted immediately after incubation and 24 or 48 h later. Data are presented as means ± SD from four individual experiments, each repeated at least in five times. * *p* < 0.05 vs. control, # *p* < 0.05 vs. drug used alone. Values are presented as a fold increase of the control.

**Figure 12:**
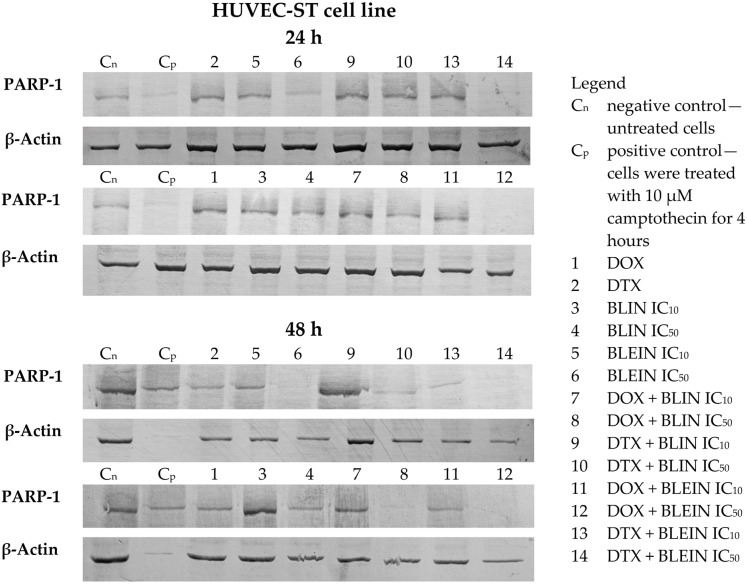
The expression of PARP-1 and β-Actin in HUVEC-ST cells incubated for 24 h with IC_10_ or IC_50_ concentration of BLIN or BLEIN alone or in combination with anticancer drugs (doxorubicin or docetaxel). The proteins were separated by SDS-PAGE, and their levels were analyzed by Western blot 24 and 48 h after incubation.

Baicalin and its aglycone baicalein induced PARP degradation in both cell types. A statistically significant increase in the cleaved form of PARP was observed after treatment with higher concentrations of flavonoids during longer postincubation times (Figure 11a). The highest amount of cleaved PARP, nearly two times higher than in control cells, was observed in HUVEC-ST cells 24 and 48 h after incubation with IC_50_ concentration of baicalein. Additionally, as shown in the blots (lanes 4 and 6) in Figure 12, associated decreased PARP-1 protein expression was observed 24 h after incubation with IC_50_ concentration of baicalein and 48 h after incubation with IC_50_ concentration of baicalin or baicalein.

**Figure 13:**
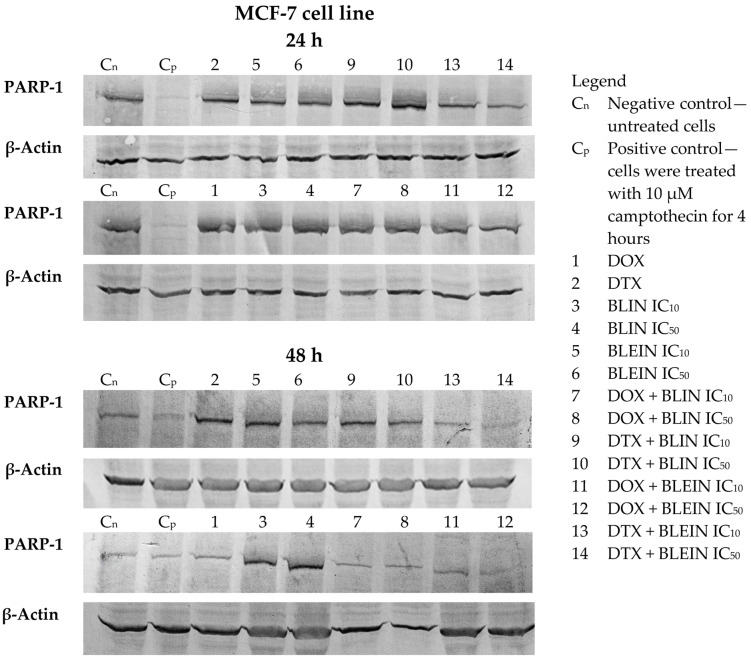
The expression of PARP-1 and β-Actin in MCF-7 cells incubated for 24 h with IC_10_ or IC_50_ concentration of BLIN or BLEIN alone or in combination with anticancer drugs (doxorubicin or docetaxel). The proteins were separated by SDS-PAGE, and their levels were analyzed by Western blot 24 and 48 h after incubation.

Forty-eight hours after incubation with doxorubicin, a statistically significant increase in the cleaved form of PARP (Figure 11b) and a decrease of PARP-1 protein expression (Figure 12 and Figure 13, lane 1) was observed in MCF-7 and HUVEC-ST cells. In the case of MCF-7 cells, only doxorubicin combined with a high concentration of baicalein during the longest postincubation time caused a significant increase in the cleaved form of PARP. None of the investigated concentrations of baicalin and its aglycone baicalein enhanced PARP cleavage induced by doxorubicin in cancer cells. However, the western blot analysis indicated decreased PARP-1 protein expression during the longest postincubation time after the treatment with doxorubicin in both investigated combinations with flavonoids (Figure 13, lanes 7, 8, 11 and 12). In contrast, the influence of investigated flavonoids on doxorubicin activity was diverse in endothelial cells. The preincubation of HUVEC-ST cells with low concentrations of baicalin and baicalein, during the longest postincubation time, diminished PARP cleavage induced by anthracycline, in the case of baicalin even to the level of control. On the other hand, high concentrations of baicalin and baicalein, especially during the longest postincubation time, enhanced PARP cleavage induced by doxorubicin. After treatment with these combinations, a statistically significant increase in the cleaved form of PARP (Figure 11b) and a decrease in PARP-1 protein expression (Figure 12, lanes 8 and 12) was observed. The twice higher than in control cells amount of cleaved PARP was observed in HUVEC-ST cells 48 h after incubation with combinations of doxorubicin with an IC_50_ concentration of baicalein.

During the longer postincubation times (24 and 48 h) after treatment with docetaxel alone, a statistically significant increase in the cleaved form of PARP (Figure 11c) was observed in MCF-7 and HUVEC-ST cells. The decrease of PARP-1 protein expression was observed only in endothelial cells 48 h after the treatment with taxane (Figure 12, lane 2). In cancer cells, a significant increase in the amount of cleaved PARP was observed 24 and 48 h after the treatment with docetaxel with baicalin (both concentrations) and 48 h after the treatment with docetaxel with baicalein (both concentrations). In all of the investigated postincubation times and compound combinations, the PARP cleavage was the most intense (a 2.5-fold increase to control) in MCF-7 cells after incubation with docetaxel with an IC_50_ concentration of baicalin. Baicalin at a high concentration significantly enhances taxane activity in breast cancer cells. However, as shown in the blots (lanes 13 and 14) in Figure 13, decreased PARP-1 protein expression was observed 48 h after incubation with docetaxel with IC_10_ and IC_50_ concentrations of baicalein. According to HUVEC-ST cells, the taxane, combined with flavonoids (both concentrations), induced PARP cleavage during the longer postintubation times. Only baicalein at an IC_50_ concentration (24 h after the incubation) significantly enhanced docetaxel activity. In the rest of the investigated combinations, the amount of cleaved PARP in HUVEC-ST cells was similar to those treated only with taxane. The Western blot analysis showed decreased PARP-1 protein expression in endothelial cells 24 h after incubation with DTX in combination with an IC_50_ concentration of baicalein and 48 h after incubation with DTX in combination with an IC_50_ concentration of baicalin and both concentrations of baicalein (Figure 12, lanes 10, 13, and 14).

## 3. Discussion

Accumulating evidence suggests that combining conventional chemotherapy with baicalin or baicalein might be beneficial for cancer treatment. These compounds exert synergistic effects with anticancer drugs by enhancing their efficacy at low doses, minimizing potential adverse effects, and overcoming multi-drug resistance [24].

Studies have shown that baicalin and baicalein synergistically improve cisplatin’s anticancer ability against various resistant cancer cells [25,26,27]. Flavonoids promoted and potentiated the apoptosis rate in cisplatin-treated cells by inhibiting the PI3K/AKT/NF-κB pathway and induction of apoptosis by diverse mechanisms, including activating caspases [28,29]. Moreover, baicalin and baicalein reduced cisplatin resistance by inhibiting the expression of genes involved in drug resistance, such as MRP1 [30] and Bcl-2, and via the Akt/mTOR and Nrf2/Keap 1 pathway [26]. In addition, baicalin and baicalein can also help decrease the side effects of cisplatin treatment by protecting the liver from damage [31] and reducing inflammation [25]. Li et al. [32] confirmed that baicalein combined with fluorouracil (5-FU) or epirubicin significantly induced the apoptosis and autophagy of multi-drug resistant hepatocellular carcinoma cells (HCC) known to be resistant to the anticancer drugs 5-FU and epirubicin. Flavonoids reversed the multi-drug resistance by inhibiting the P-glycoprotein mediated drug efflux and enhancing the intracellular accumulation of chemotherapy drugs.

Baicalin and its aglycone baicalein show promise in overcoming drug resistance and enhancing the effectiveness of chemotherapy in breast cancer treatment. Recent findings suggest that baicalein can restore sensitivity in tamoxifen-resistant breast cancer cells, potentially offering a new approach to overcome resistance [33]. The flavonoid significantly increased the sensitivity of resistant cells to tamoxifen by inhibiting cell proliferation and inducing apoptosis via the inhibition of HIF-1α, a protein that promotes cancer growth. In Hua et al.’s [34] study, baicalein sensitizes a subtype of the refractory breast cancers with resistance to chemotherapy and poor prognosis to doxorubicin via the autophagy-mediated down-regulation of CDK1. Zeng et al. [35] found that baicalin sensitized breast cancer cells to docetaxel by suppressing the expression of survivin/Bcl-2. Baicalin, in combination with doxorubicin, elevated the chemosensitivity of MDA-MB-231 and MCF-7 breast cancer cells compared to single drug treatment. The flavonoid enhanced drug cytotoxicity toward breast cancer cells by increasing the level of intracellular ROS and the further upregulation of oxidative-stress-mediated mitochondria-dependent apoptosis [36].

Furthermore, flavonoids demonstrate selectivity in their action. They attenuate drug side effects without compromising their antitumor activity and preserving their therapeutic efficacy. Due to its antioxidant properties, baicalein effectively scavenged reactive oxygen species generated by doxorubicin [37]. The flavonoid restored myocardial non-enzymatic and enzymatic antioxidants and the down-regulated intrinsic and NF-κB regulated apoptotic pathway associated with doxorubicin-induced cardiomyocyte cell death. By suppressing NF-κB signaling, baicalein reversed doxorubicin-mediated inflammation within the myocardium [38]. Additionally, baicalein preserved cardiac function by maintaining tissue integrity and diminishing the elevation of serum biomarkers such as creatine kinase-MB isoenzyme (CK-MB), lactate dehydrogenase (LDH), aspartate aminotransferase (AST), and alanine aminotransferase (ALT), indicative of myocardial damage [39]. Similar to baicalein, baicalin also significantly protects against doxorubicin’s cardiotoxicity. It reduces inflammation, manages oxidative stress, and potentially helps cell repair to preserve heart tissue and cardiac fibrosis [40].

**Figure 14:**
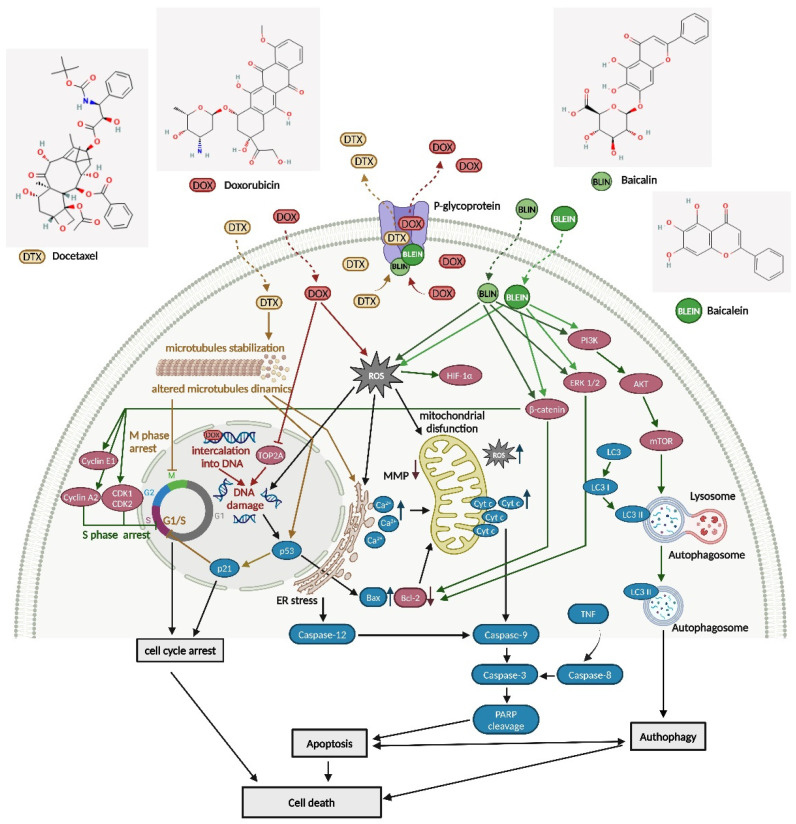
A schematic diagram illustrating the structures of the investigated molecules and the cellular and molecular targets of anticancer drugs docetaxel and doxorubicin, as well as flavonoids baicalin and baicalein, in association with their anticancer activity. (Boxes in dark pink indicate downregulation of expression and boxes in blue indicate upregulation of expression). Created with BioRender.com.

Based on recent research findings [41,42,43,44,45,46,47], a hypothetical scheme (Figure 14) has been proposed to demonstrate cellular and molecular targets of docetaxel and doxorubicin, as well as baicalin and baicalein, in relation to their anticancer activity. While doxorubicin targets DNA replication and docetaxel disrupts microtubule dynamics, baicalin and baicalein act through a more complex network of cellular processes. These flavonoids can interfere with signaling pathways involved in cell growth and survival, potentially reversing cancer multidrug resistance. As shown in Figure 14, baicalin and baicalein affect other molecular targets than doxorubicin or docetaxel. Thus, they can provide benefits in overcoming cancer drug resistance mechanisms by decreasing the efflux of anticancer drugs, reducing DNA damage repair, and activating apoptosis and autophagy.

In the first stage of our study, we analyzed the cytotoxicity of baicalin and its aglycone baicalein towards the estrogen-dependent breast cancer cell line MCF-7 and the human umbilical vein endothelial cell line HUVEC-ST. We assessed cytotoxicity using methods based on various parameters that indicate disturbances in cell physiological processes, such as changes in the activity of redox enzymes (MTT assay and resazurin reduction assay) and changes in the integrity of the cell membrane (neutral red uptake assay).

After a 24-h treatment with flavonoids, a significant reduction in the number of living cells was observed in both cancer and endothelial cell lines, regardless of the applied method. For the MCF-7 cell line, the inhibitory concentration IC_50_ of baicalin and baicalein was calculated to be 250 ± 10.5 µmol/L and 95 ± 4.8 µmol/L, respectively. These results are consistent with other studies, indicating that baicalin and baicalein exhibit cytotoxic potential toward various cancer cell lines. The concentration of flavonoids required to reduce cell viability by 50% (IC_50_) depends on the type of cell line and treatment time (usually 24–72 h) and ranges from 20 to 200 µmol/L [20].

Liu et al. [48] demonstrated that baicalein significantly inhibited MCF-7 cell growth in a dose-dependent manner with the IC_50_ value of 85.07 ± 1.26 µmol/L. According to baicalin, Wang et al. [49] did not observe any inhibitory effect on MCF-7 cell proliferation after treatment with 5–100 µmol/L of baicalin for 72 h, which suggests that higher concentrations of flavonoid are required to reduce cell viability by 50%. Interestingly, So et al. [50,51] have reported that baicalein can inhibit the proliferation of estrogen-dependent and estrogen-independent breast cancer cells. These results suggest that the antiproliferative activity of flavonoid is independent of the presence of estrogen receptors on the cell surface, which excludes the possibility of competition between baicalein and estradiol for binding via the estrogen receptor. Besides, baicalein can inactivate the G protein-coupled estrogen receptor (GPR30) signaling pathway and decrease the phosphorylation of AKT and ERK 1/2, the tyrosine phosphorylation of AKT and ERK 1/2, and the tyrosine phosphorylation of epidermal growth factor receptor (EGFR) in breast cancer cells, leading to the suppression of the migration and invasion of cancer [52]. The mentioned properties make baicalein helpful in supporting the treatment of receptor-negative cancers characterized by a worse prognosis and poor response to therapy.

The calculated inhibitory concentration IC_50_ of baicalin and baicalein for the HUVEC-ST cell line was, respectively, 167 ± 6.7 µmol/L and 115 ± 2.6 µmol/L. Flavonoids induced cytotoxicity only at high concentrations. No significant variation in cell viability was observed for the lower doses. Similarly, Liu et al. [53] demonstrated that treatment with baicalin or baicalein for 48 h led to a dose-dependent decrease in HUVEC cell viability. At the highest concentration tested, 50 µmol/L, the percentages of viable HUVECs after treatment with baicalin and baicalein were 61% ± 8% and 37% ± 3% of the control, respectively. Notably, due to the ability to modulate cell-signaling pathways, baicalin and baicalein exhibit diverse effects on the human umbilical vein endothelial cells. On the one hand, they protect HUVECs from damage caused by various factors, including oxidative stress, inflammation, and toxins, due to their ability to scavenge free radicals, reduce inflammation, and modulate signaling pathways [54,55]. This property might help prevent vascular endothelial dysfunction, which is an essential contributor to atherosclerosis and cardiovascular diseases (CVD) (e.g., coronary artery disease, carotid artery disease, peripheral artery disease, and ischemic stroke) [56]. On the other hand, in an in vitro study using human umbilical vein endothelial cells (HUVECs), baicalin and baicalein were shown to reduce the proliferation, migration, and tube formation of HUVECs, crucial steps in the angiogenesis process [53,57]. These anti-angiogenic effects result from the suppression of the expression of pro-angiogenic factors, such as vascular endothelial growth factor (VEGF) [58], and the induction of the expression of anti-angiogenic factors, such as thrombospondin-1 (TSP-1) [54,59].

According to our research, baicalein had a more powerful cytotoxic potential towards both cell lines than its glucuronide baicalin. This is because baicalein has a smaller size and high lipophilicity, contributing to fast absorption and an improved ability to penetrate cells [60]. The presence of a hydroxyl group at the C-6 position in the A ring of the baicalein molecule significantly determines its high cytotoxicity. Glycosides having a sugar residue in their molecule are characterized by substantially lower activity [61].

Further analysis focused on investigating baicalin and baicalein’s effect on the cytotoxicity of anticancer drugs and the type of interaction in flavonoid–drug combinations. Both investigated anticancer drugs revealed expected cytotoxicity towards breast cancer cells; the calculated IC_50_ value of doxorubicin was 3 µmol/L, and that of docetaxel was 0.1 µmol/L. Additionally, whereas doxorubicin showed similar cytotoxicity towards MCF-7 and HUVEC-ST cells, the highest tested docetaxel concentration (6 µmol/L) caused only a 20% decrease in the survival of human endothelial cells. Preincubation with high concentrations of baicalin or baicalein (IC_50_) for 22 h strongly enhances doxorubicin anticancer activity toward breast cancer cells. Flavonoids showed a strong synergism with doxorubicin. The drug, at a concentration of 3 µmol/L combined with flavonoids, provoked a more significant decrease in cell viability than when used alone. The viable cells decreased from 54% to 21% after preincubation with baicalin and from 54% to 10% after preincubation with baicalein. Moreover, both investigated flavonoids’ high concentrations (IC_50_) significantly increased doxorubicin influx into breast cancer cells. The amount of doxorubicin in breast cancer cells preincubated with baicalin or baicalein was over two times higher than in cells not treated with flavonoids. Both flavonoids also acted synergistically with docetaxel, decreasing the number of viable MCF-7 cells by about half. In the HUVEC-ST cell line, the preincubation with baicalein increased the cytotoxicity of doxorubicin. The CI index calculated for the combination of DOX IC_50_ + BLEIN IC_50_ was 0.68010, indicating a synergistic relationship. Doxorubicin accumulation increased for the entire study period after preincubation with baicalein’s IC_50_ concentration. Additionally, baicalein potentiates the cytotoxicity of the taxane against endothelial cells when combined with anthracycline. However, baicalin did not affect doxorubicin and docetaxel cytotoxicity in HUVEC-ST cells. The significant enhancement of doxorubicin influx into HUVEC-ST cells preincubated with baicalin was observed only at the beginning of the measurement. The highest CI index value of 3.1 was observed when combining docetaxel with baicalin at IC_50_ in HUVEC-ST cells, indicating the most potent antagonistic effect among the combinations tested.

These findings offer compelling evidence that high concentrations of investigated flavonoids exhibit a strong synergism with doxorubicin and docetaxel, especially in breast cancer cells. Moreover, they caused a significant increase in doxorubicin levels inside the cells. The increased drug retention may be due to the ability of flavonoids to modify the activity of transport proteins, which are part of the transmembrane ABC transporters [62]. In a study conducted by Boumendjel et al. [63], flavonoids (aglycones), with a hydroxyl group at the C-3 position of the A ring and a double bond between the C2–C3 atoms in the C ring, show a higher affinity to the hydrophilic glycoprotein-P domain (nucleotide-binding domain, NBD) on the cytoplasmic side of the cell membrane. Glycosides do not exhibit the ability to bind to the NBD domain of glycoprotein-P but, together with aglycones, can inhibit the activity of the protein MRP1, whose overexpression accompanies anthracycline resistance [64]. Baicalein has been shown to inhibit the activity of MRP2 and MRP1 proteins at a concentration of 25 µmol/L [65]. The increase in the intracellular concentration of doxorubicin is a highly desirable effect in cancer cells because of the possibility of achieving the correct drug concentration in target cells and comparable therapeutic effects at significantly reduced doses of the cytostatic agent. However, there is strong concern regarding their impact on healthy cells. They may inadvertently increase the efflux of essential drugs from healthy cells, which could lead to unintended side effects. This may also result in increased drug toxicity due to the insufficient uptake and retention of the drug in its target site within healthy cells.

Abnormalities in programmed cell death underlie both the development of cancer and the resistance of cancer cells to anticancer therapies. These abnormalities arise from genetic alterations, such as the overexpression or mutation of proto-oncogenes, as well as the ability of cancer cells to evade the mechanisms of programmed cell death. This evasion leads to increased cell viability, prolonged cell lifespan, the persistence of acquired mutations, disruptions in cell cycle regulation, and resistance to anticancer drugs. There are two pathways by which apoptosis can be triggered: the extrinsic pathway (death-receptor-mediated pathway) and the intrinsic pathway (mitochondrial-mediated pathway). The intrinsic apoptotic pathway involves a change in mitochondrial permeability by Bcl-2-related proteins, leading to the release of cytochrome c. This activates the caspase cascade, resulting in cell apoptosis [66]. The altered susceptibility of cancer cells to apoptosis significantly limits the effectiveness of anthracycline- and taxane-based breast cancer therapies [67]. Therefore, it is crucial to develop strategies that enhance the proapoptotic effect of chemotherapeutics to improve the efficacy of anticancer treatments.

Numerous studies reported that baicalin and baicalein can induce apoptosis in various tumors through both extrinsic and intrinsic pathways. The anticancer effects of baicalin and baicalein are diverse, including the induction of apoptosis and triggering of autophagy. Both flavonoids can cause the cleavage of mitochondrial-dependent caspases and upregulation of p53, Bax, and cytochrome C, which leads to a loss of mitochondrial membrane potential and a reduction in Bcl-2 levels in cell lines with different degrees of sensitivity [24]. Therefore, in this study, further analysis is focused on the impact of baicalin and baicalein on the proapoptotic activity of anticancer drugs by measuring changes in membrane lipid asymmetry related to induction apoptosis and necrosis. As the mitochondrial membrane potential (ΔΨm) plays a crucial role in the induction of apoptosis, changes in (ΔΨm) were also measured.

Our results demonstrate that baicalin and baicalein can induce apoptosis and necrosis in a concentration and time-dependent manner in both MCF-7 and HUVEC-ST cells. Changes observed in both cell types were similar. The induction of apoptosis was associated with a decrease in mitochondrial membrane potential (ΔΨm). Interestingly, the mitochondrial membrane potential (ΔΨm) of HUVEC-ST cells treated with baicalin and baicalein did not decrease substantially. Both baicalin and baicalein showed the ability to enhance doxorubicin’s and docetaxel’s anticancer activity, but the effect varies depending on the cell type and flavonoid concentration. The longest post-incubation (48 h) showed an increase in the enhancement of anthracycline proapoptotic activity towards breast cancer cells. While doxorubicin alone induced apoptosis only in 12% of MCF-7 cells, the percentage of AV+/PI− cells that underwent apoptosis after treatment with the combination with baicalin or baicalein at IC_50_ concentration was 34% and 21%, respectively. The mitochondrial membrane potential (ΔΨm) of MCF-7 cells was reduced to 57% after the treatment with anthracycline. Combining doxorubicin with baicalin or baicalein at high concentration decreased (ΔΨm) to 31% and 20%, respectively. Only baicalin enhanced taxane proapoptotic activity towards cancer cells, but the fraction of cells that exhibit significant changes in membrane lipid asymmetry—Annexin-V-positive—mainly consisted of AV+/PI+ cells, suggesting that this population predominantly consisted of cells in late-stage apoptosis. Preincubating cancer cells with baicalin or baicalin at a high concentration (IC_50_) significantly enhanced the reduction of mitochondrial membrane potential caused by taxane. In the case of the HUVEC-ST cell line, high concentrations (IC_50_) of baicalein enhanced doxorubicin’s proapoptotic activity and enhanced the reduction of mitochondrial membrane potential caused by anthracycline. During longer postincubation times, baicalein almost doubled the number of apoptotic cells compared to doxorubicin alone. In contrast, baicalin did not enhance the proapoptotic activity of doxorubicin or the reduction of mitochondrial membrane potential caused by the drug. Surprisingly, a flavonoid slightly decreased doxorubicin’s proapoptotic activity during the longest postincubation time. The baicalein at high concentration also enhanced docetaxel’s proapoptotic activity, increasing the number of AV+/PI+ cells. Baicalin at both high and low concentrations did not affect docetaxel’s activity. Neither baicalin nor baicalein at any concentration significantly impacted docetaxel-induced mitochondrial membrane potential changes.

One of the main molecular mechanisms of anthracyclines’ cytostatic and cytotoxic activity is DNA damage to cancer cells, whose genetic material is susceptible to the action of mutagenic and genotoxic factors due to excessive cell proliferation. This DNA damage can occur directly when the drug molecules interact with DNA or indirectly through reactions involving free radicals generated during anthracycline redox reactions [68]. Meanwhile, taxanes inhibit microtubule depolymerization, thereby preventing mitosis and the division of genetic material [10]. Both drug classes can induce DNA damage, one of the factors that can trigger the mitochondrial pathway of apoptosis. During apoptosis, the cell death program is activated irreversibly, leading to the destruction of the nucleus, DNA fragmentation, cytoskeletal and protein degradation, chromatin condensation, and the formation of apoptotic bodies.

Our study revealed that high concentrations of baicalin and baicalein (IC_50_) can cause DNA damage in MCF-7 and HUVEC-ST cells. Both flavonoids enhanced damages caused in breast cancer cells by drugs alone, but baicalein seems to be more potent. In endothelial cells, baicalin and baicalein have mixed effects on the genotoxicity of doxorubicin and docetaxel, depending on the postincubation times. They enhance drug genotoxicity immediately or 24 h after incubation, while 48 h after incubation, they tend to reduce DNA damage. Only the combination of taxane with baicalein (IC_50_) strongly enhances taxane genotoxicity, especially 48 h after incubation.

Poly(ADP-ribose)-1 polymerase is one of the most important enzymes responsible for maintaining genome stability and properly functioning transcription factors. In normal cells, PARP-1 activity is low, but it increases rapidly under genotoxic or oxidative stress conditions. Exposure to DNA-damaging agents can cause a 10- to 500-fold increase in PARP-1 levels. Excessive activation of poly(ADP-ribose)-1 polymerase leads to an energy deficiency in the cell and triggers programmed cell death. Therefore, the proteolytic degradation of PARP-1 is considered one of the early events in the onset of apoptosis [69].

We have observed that baicalin and baicalein cause PARP degradation in both cell types. The highest amount of cleaved PARP was observed in HUVEC-ST cells after 24 and 48 h of incubation with IC_50_ concentrations of baicalein. Doxorubicin also induced PARP cleavage in both cell types, but baicalin and baicalein did not enhance this effect in MCF-7 cells. In HUVEC-ST cells, low concentrations of baicalin and baicalein reduced PARP cleavage induced by doxorubicin, while high concentrations enhanced it. Docetaxel induced PARP cleavage in both cell types, and baicalin significantly enhanced this effect in MCF-7 cells. Generally, flavonoids did not enhance taxane activity in HUVEC-ST cells.

## 4. Materials and Methods

### 4.1. Materials

#### 4.1.1. Chemicals

Culture media (OptiMEM and DMEM), fetal bovine serum (FBS), antibiotics 10 U/mL penicillin, and 50 μg/mL streptomycin and trypsin-EDTA were obtained from Gibco (Thermo Fisher Scientific, Waltham, MA, USA). Doxorubicin and docetaxel were purchased from Sequoia Research Products Ltd. (Pangbourne, UK).

Baicalein (5,6,7-Trihydroxyflavone); baicalin (baicalein 7-*O*-β-d-glucuronide); MTT; Neutral Red; Resazurin; 5,5′,6,6′-Tetrachloro-1,1′,3,3′tetraethylbenzimidazolo-carbocyanine iodide (JC-1); 4′,6-Diamidino-2-phenylindole dihydrochloride (DAPI); 3-Amino-7-dimethylamino-2-methyl-phenazine hydrochloride (Neutral red); LMP agarose type XI; NMP agarose type I; RIPA buffer; and secondary antibodies conjugated with alkaline phosphatase were purchased from Sigma-Aldrich (St. Louis, MO, USA). The FITC Annexin V Apoptosis Detection Kit II was purchased from BD Pharmingen (San Diego, CA, USA). The Cleaved PARP-1 In-Cell ELISA Kit was purchased from Thermo Fisher Scientific (Waltham, MA, USA). Mouse monoclonal antibodies specific to PARP1 were purchased from Santa Cruz Biotechnology, Inc. (Dallas, TX, USA). Unless otherwise indicated, all of the other chemicals were purchased from Avantor Performance Materials Poland S.A. (Gliwice, Poland).

#### 4.1.2. Cell Culture

The estrogen-receptor-positive MCF-7 human breast cancer cell line (ATCC, Manassas, VA, USA) was grown in Dulbecco’s modified Eagle’s medium (DMEM) enriched with 10% fetal bovine serum and supplemented with antibiotics (10 U/mL penicillin and 50 μg/mL streptomycin). The immortalized human umbilical vein endothelial cell line HUVEC-ST was obtained from Prof. G. Bartosz (Department of Molecular Biophysics, University of Lodz, Poland) and grown in OptiMEM medium enriched with 3.5% fetal bovine serum and supplemented with antibiotics (10 U/mL penicillin and 50 μg/mL streptomycin). Both cell lines were cultured as a monolayer under the standard conditions (an atmosphere of 5% CO_2_ and 95% air, 37 °C, and 100% humidity). In all of the experiments, cells in the logarithmic phase of growth were used. Cells at 80% confluence were carefully removed with trypsin/EDTA, washed with fresh phosphate-buffered saline (PBS), and assessed for viability using the trypan blue assay.

#### 4.1.3. Treatment Conditions

Baicalein (BLEIN) and baicalin (BLIN) were dissolved in DMSO. Doxorubicin (DOX) was dissolved in water, and docetaxel (DTX) was dissolved in ethanol. The final concentration of the DMSO at the highest investigated concentration of BLIN/BLEIN did not exceed 0.2%; similarly, the final concentration of the ethanol at the highest investigated DTX concentration did not exceed 0.3%.

Unless otherwise indicated, in experiments, cells were seeded at an appropriate density for the specific assay into 96-well plates or Petri dishes. They were then incubated in standard culture conditions, which included a temperature of 37 °C, 100% relative humidity, and a 5% CO_2_ incubator. The schedule for incubation was followed accordingly: (a) 24 h with IC_10_ or IC_50_ concentration of BLIN or BLEIN, (b) 2 h with IC_50_ concentration of DOX or DTX, and (c) 22 h with IC_10_ or IC_50_ concentration of BLIN or BLEIN and subsequently 2 h with IC_50_ concentrations of DOX or DTX. After treatment, the cells were washed with PBS and, depending on the experiment, immediately used for measurement or cultured in a fresh medium for 24 or 48 h. Control cells were treated with a corresponding volume of PBS. Additional controls for solvents were also performed.

### 4.2. Methods

#### 4.2.1. Cell Proliferation Analysis

- MTT assay

The principle of the method is a measurement of the absorbance of formazan, a product of the reduction of the water-soluble tetrazolium salt (MTT) by NADPH-dependent cellular oxidoreductases (mainly succinate dehydrogenase) in living cells. Formazan is insoluble in water and precipitates as purple crystals. The amount of formazan is proportional to the number of living and metabolically active cells.

After treatment, the cells were washed with PBS and cultured in a fresh medium for 48 h. Subsequently, the medium was aspirated, and an aliquot of 50 µL of 0.05 mg/mL MTT solution was added to each microplate well for 3 h. The formazan crystals were dissolved in dimethyl sulfoxide (DMSO), and the absorbance was measured at a wavelength of λ = 580 nm. The percentage of viable cells was calculated by comparing the absorbance value of the test samples to the absorbance value of the control, taking the absorbance of the control as 100%.

- Neutral red uptake assay

The neutral red uptake assay measures the ability of living cells to absorb and bind the supravital dye neutral red (NR) inside organelles with an acidic pH (such as lysosomes and endosomes). The dye is transported into the cells passively, and the amount of neutral red accumulated in the organelles is directly proportional to the number of living cells.

After treatment, the cells were washed with PBS and cultured in a fresh medium for 48 h. Subsequently, the medium was aspirated, and an aliquot of 100 µL of 0.05 mg/mL NR solution was added to each microplate well for 3 h. Behind this, the cells were washed, the dye was extracted in each well, and the absorbance was measured at a wavelength of λ = 540 nm. The percentage of viable cells was calculated by comparing the absorbance value of the test samples to the absorbance value of the control, taking the absorbance of the control as 100%.

- Resazurin reduction assay

The assay employs a nonfluorescent dye resazurin. Cytosolic, mitochondrial, and microsomal redox enzymes reduce resazurin to the strongly fluorescent dye resorufin. The fluorescence output correlates highly with the number of viable and metabolically active cells.

After treatment, the cells were washed with PBS and cultured in a fresh medium for 48 h. Subsequently, the medium was aspirated, and an aliquot of 100 µL of 0.0125 mg/mL resazurin solution was added to each microplate well for 3 h. Behind this, the fluorescence of resorufin was measured on a spectrofluorometer Cary Eclipse (Varian, Inc., Palo Alto, CA, USA) at λ_ex_ = 530 nm, λ_em_ = 590 nm. The percentage of viable cells was calculated by comparing the fluorescence intensity of the test samples to the fluorescence intensity of the control, taking the fluorescence of the control as 100%.

#### 4.2.2. Quantitation of Synergism and Antagonism in Flavonoid–Drug Combinations

The experiment with a non-constant combination of BLIN and BLEIN with DOX or DTX was carried out to determine the synergistic, additive, or antagonistic effect between investigated flavonoids and anticancer drugs. Both cell lines were treated for 22 h with an IC_10_ or IC_50_ concentration of BLIN or BLEIN and subsequently 2 h with DOX at concentrations 0.25, 0.5, 1, 2, 3, 4, 5, and 6 μmol/L or DTX at concentrations 0.02, 0.05, 0.1, 0.3, 0.5, 1, 2, and 4 μmol/L. Subsequently, cell viability was determined by MTT assay. The data from this experiment were analyzed using CompuSyn software v. 1.0 for drug combinations (ComboSyn, Inc., Paramus, NJ, USA). The results were presented as a combination index (CI). A CI value is a mathematical and quantitative representation of the pharmacological interplay of two drugs (CI > 1—antagonism; CI = 1—additive; and CI < 1—synergism).

#### 4.2.3. Cytometric Measurement of Doxorubicin Accumulation

The aromatic rings in the hydrophobic part of doxorubicin give the drug molecule fluorescent properties (λ_ex_ = 490 nm, λ_em_ = 590 nm). Thus, the flow cytometric measurement of drug fluorescence inside the cells allows for the monitoring of its transport rate through the cell membrane. The increase in the intensity of drug fluorescence inside the cells is highly correlated with its accumulation.

The cells were incubated for 22 h with an IC_50_ concentration of BLIN or BLEIN, then trypsinized and suspended in a culture medium. The DOX was added to the cell suspension to achieve a final concentration equal to the IC_50_ value. During one hour of incubation (temp 37 °C) with the drug, samples for cytometric measurement were taken from the cell suspension after the first 5 min and then after every 10 min. Samples were analyzed using the flow cytometer LSR^®^ II from Becton Dickinson (San Jose, CA, USA) using an argon laser with a wavelength of λ = 488 nm. Doxorubicin fluorescence intensity was calculated per single cell.

#### 4.2.4. Annexin V FITC/Propidium Iodide Double Staining Assay

This assay is based on the principle that, in viable cells, phosphatidylserine (PS) is located in the inner membrane (side facing the cytoplasm). However, upon induction of apoptosis, PS is translocated to the outer membrane (PS also appears on the necrotic cell surface). The exposed phosphatidylserine can be detected by protein Annexin V stained with FITC dye. The addition of propidium iodide (PI) enabled necrotic cells to be distinguished. Thus, this method is convenient for counting and differentiating live cells from those undergoing apoptosis or necrosis.

The analysis was performed according to the manufacturer’s protocol on cells immediately after the treatment and 24 or 48 h postincubation. Samples were analyzed using the flow cytometer LSR^®^ II from Becton Dickinson (San Jose, CA, USA) using an argon laser with a wavelength of λ = 488 nm.

#### 4.2.5. Measurement of Mitochondrial Membrane Potential

The mitochondrial membrane potential was measured with the membrane-permeant JC-1 fluorescent probe [70]. This fluorescent carbocyanine dye exhibits potential-dependent accumulation in mitochondria in two forms: monomers with a green fluorescence emission, which shifts to red fluorescence emission dimers during mitochondrial membrane depolarization. Thus, the measurement of the ratio of JC-1 dimer to monomer fluorescence can be used for estimating changes in mitochondrial membrane potential (ΔΨm).

After the treatment, the cells were immersed in Hank’s buffered salt solution (HBSS) that had a 5 µmol/L JC-1 fluorescent probe. The cells were then incubated in complete darkness for 30 min at 37 °C. After incubation, the cells were washed to remove the dye and suspended in fresh HBSS solution. Finally, the fluorescence intensity was measured using a Cary Eclipse spectrofluorometer (Varian, Inc.) at λ_ex_/_em_ = 475 nm/530 nm for monomers and at λ_ex_/_em_ = 535 nm/590 nm for dimers. Subsequently, the ratio of the fluorescence intensities at 530 nm/590 nm was calculated. The mitochondrial potential in both cell lines was measured immediately after incubation with investigated compounds. The results are expressed as a percentage of the control, considered 100%.

#### 4.2.6. The Comet Assay

The comet assay, also known as the single-cell gel electrophoresis assay, is a highly sensitive and versatile method for measuring single- and double-stranded DNA breaks. The assay uses an electric field to move negatively charged DNA fragments through an agarose gel. The amount of migration reflects the level of DNA damage in the cells.

The analysis was performed under alkaline conditions, according to the method of Singh et al. [71], on cells immediately after the treatment and 24 or 48 h postincubation. A suspension of cells was mixed with low-melting-point agarose (LMP type XI) and spread onto a microscope glass slide previously coated with standard melting point agarose (NMP type I). After 2 h of cells lysis in a buffer containing (2.5 mol/L NaCl, 100 mmol/L Na_2_-EDTA, 10 mmol/L TRIS, 1% Triton X-100), DNA unwinding and electrophoresis were carried out in a buffer containing (1 mmol/L Na_2_-EDTA, 300 mmol/L NaOH) and (29 V, 30 mA). Before analysis, the slides were stained with DAPI solution (2 μg/mL). One hundred randomly chosen cells were analyzed on each slide using a fluorescence microscope Zeiss Axio Scope A1 (Carl Zeiss, Oberkochen, Germany) with a video camera connected to the image-analysis system Lucia-Comet v. 7.60 (Laboratory Imaging, Praha, Czech Republic).

#### 4.2.7. Cleaved PARP ELISA Assay

The expression of the protein was monitored using a target-specific primary antibody against an 85 kDa stable fragment of PARP proteolysis and a horseradish peroxidase-conjugated detection reagent.

The amount of cleaved PARP was measured in cells according to the manufacturer’s protocol immediately after the treatment and after 24 or 48 h postincubation. The absorbance was measured at a wavelength of λ = 450. Additionally, the whole cell staining was performed with Janus Green dye, and the absorbance was measured at a wavelength of λ = 615. In order to normalize results and eliminate differences in cell numbers in various wells, the A450 values were divided by A615 from corresponding wells. Subsequently, the values were calculated as a fold increase, which means the ratio of an increased amount of cleaved PARP to the amount of cleaved PARP in control cells.

#### 4.2.8. Western Blot Detection of PARP Cleavage

After the treatment, cells were lysed at 4 °C for 20 min in a RIPA buffer containing protease inhibitors. The supernatants were collected after centrifugation, and the protein concentration was determined using the Lowry et al. [72] method. Each lane was loaded with approximately 25 µg of protein, and electrophoretic separation of the probes was carried out by 8% sodium dodecyl sulfate-polyacrylamide gel electrophoresis (SDS-PAGE). The probes were then transferred to Immobilon P, as described by Towbin et al. [73]. To prevent non-specific antibody binding, the membranes were blocked in 5% non-fat dry milk in TBST buffer for 1 h at room temperature. Next, a specific primary antibody that recognizes a protein of interest, namely, mouse monoclonal antibodies specific to PARP1 (1:1000 dilution) in a TBST buffer, was applied overnight in a cold room. After washing away unbound antibodies, the membranes were incubated with anti-mouse secondary antibodies conjugated with alkaline phosphatase in TBST (1:5000 dilution) for 2 h at room temperature. The membranes were then washed several times with TBST. The proteins were made visible by being exposed to a substrate solution prepared according to Leary et al. [74]. The change in the expression of the tested proteins was determined by analyzing the signal intensity and densitometry using ImageJ 1.48v software (National Institutes of Health, Bethesda, MD, USA).

#### 4.2.9. Statistical Analysis

The data’s normality was assessed using the Shapiro–Wilk test, and variance homogeneity was evaluated with Levene’s test. Differences between groups were determined through a one-way ANOVA and post hoc Tukey test. The results represent mean ± standard deviation (SD). Statistical significance was accepted at *p* < 0.05. The statistical analysis was conducted with Statistica v. 13.1 (StatSoft Polska, Krakow, Poland). The statistical analysis of the differences between survival curves obtained for MCF-7 and HUVEC-ST cells treated with anticancer drug alone and in combination with flavonoid was performed using the GraphPad Prism Software v. 5.04 for Windows (Boston, MA, USA). This analysis aimed to compare the fits of separate curves to each data set with a single curve fit to all the data sets. It examined whether there is evidence that the pretreatment with flavonoids did anything to shift the basal anticancer drug curves. The statistical significance indicates that one curve cannot adequately fit all of the data sets. Thus, there is a difference between the survival curve obtained for anticancer drugs alone and in combination with flavonoid.

## 5. Conclusions

This study aimed to investigate the potential of baicalin and baicalein in enhancing the anticancer activity of doxorubicin and docetaxel while assessing their toxicity towards both cancer and endothelial cells. The results showed that baicalein generally exhibited more potent cytotoxic and synergistic effects than baicalin in both cancer and endothelial cells. Particularly, MCF-7 cancer cells were found to be more susceptible to these compounds, as well as doxorubicin and docetaxel. Both flavonoids exhibited synergism with doxorubicin and docetaxel in breast cancer cells, significantly lowering cell viability, which suggests the potential for targeted cancer therapy. This synergy appears to involve increased doxorubicin accumulation within cancer cells, possibly due to flavonoid interaction with transport proteins. Baicalin and baicalein enhanced the proapoptotic activity of doxorubicin and docetaxel in breast cancer cells, evidenced by changes in membrane lipid asymmetry and mitochondrial membrane potential. Only baicalein enhanced the proapoptotic activity of doxorubicin in endothelial cells, while both flavonoids displayed mixed effects on docetaxel depending on concentration and time. Baicalin and baicalein induced DNA damage in both cell lines, potentially amplifying the effects of chemotherapeutics in cancer cells. However, their impact on doxorubicin and docetaxel genotoxicity in endothelial cells varied. Both flavonoids induced PARP cleavage in both cell types, further suggesting their potential to promote apoptosis. This study highlights the potential use of baicalin and baicalein as adjuvants to improve the efficacy of doxorubicin and docetaxel in breast cancer treatment. However, further research is required to assess their impact on other cell types relevant to chemotherapy toxicity and evaluate the efficacy and safety of these flavonoid–drug combinations in preclinical models. Moreover, bioavailability limitations must be overcome to unlock their therapeutic potential.

## Figures and Tables

**Table 1:** Baicalin and baicalein inhibitory concentrations IC_10_ and IC_50_ (µmol/L).

Inhibitory Concentration (µmol/L)		
	MCF-7 cell line	HUVEC-ST cell line
baicalin IC_10_	36 ± 3.4	101 ± 10.7
baicalin IC_50_	250 ± 10.5	167 ± 6.7
baicalein IC_10_	9 ± 0.7	75 ± 1.8
baicalein IC_50_	95 ± 4.8	115 ± 2.6

**Table 2:** Combination index (CI) values calculated on the basis of survival curves shown in Figure 3 and Figure 4.

Combination Index (CI)				
	MCF-7 cell line		HUVEC-ST cell line	
drug + flavonoid	DOX IC_50_	DTX IC_50_	DOX IC_50_	DTX IC_50_
baicalin IC_10_	1.15812	0.96524	2.91523	0.99482
baicalin IC_50_	0.64347	0.54206	1.79361	3.13654
baicalein IC_10_	1.07317	1.07966	0.87213	0.96110
baicalein IC_50_	0.34117	0.62760	0.68010	0.44278

**Table 3:** Examples of photographs taken during the comet assay. The photographs were captured using a fluorescence microscope Zeiss Axio Scope A1 (Carl Zeiss, Germany) with a video camera connected to the image-analysis system Lucia-Comet v. 7.60 (Laboratory Imaging, Praha, Czech Republic).

(a) DOX (24 h) MCF-7 cell line	(b) DOX (48 h) HUVEC-ST cell line	(c) DTX (24 h) MCF-7 cell line
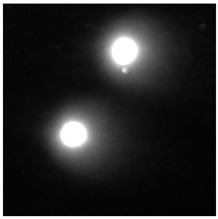	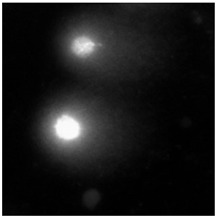	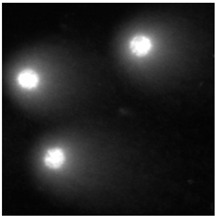
(d) DTX (24 h) HUVEC-ST cell line	(e) BLIN IC_50_ (24 h) MCF-7 cell line	(f) BLIN IC_50_ (24 h) HUVEC-ST cell line
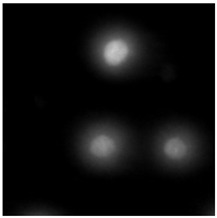	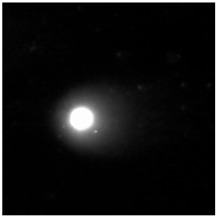	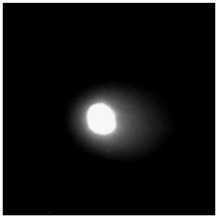
(g) DTX+ BLEIN IC_50_ (0 h) MCF-7 cell line	(h) DOX+ BLIN IC_50_ (48 h) HUVEC-ST cell line	(i) Control cells MCF-7 cell line
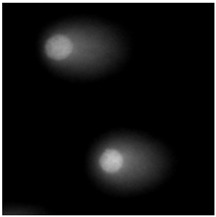	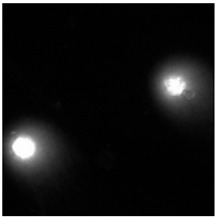	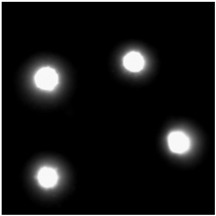

## Data Availability

The data presented in this study are available on request from the corresponding author.

## Abbreviations

AKT—protein kinase B family; Bax—Bcl-2-associated X protein; Bcl-2—B-cell leukemia/lymphoma 2 protein; CDK1—cyclin-dependent kinase 1; Cyt c—cytochrome c; ERK 1/2—extracellular signal-regulated kinases 1 and 2; HIF-1α—hypoxia-inducible factor 1-alpha; Keap 1—Kelch-like ECH-associated protein 1; LC3—microtubule-associated protein 1A/1B-light chain 3; MMP—mitochondrial membrane potential (ΔΨm); MRP1—multidrug resistance-associated protein 1; MRP2—multidrug resistance-associated protein 2; mTOR—mammalian target of rapamycin; NF-κB—nuclear factor kappa-light-chain-enhancer of activated B cells; Nrf2—nuclear factor (erythroid-derived 2)-like 2; p21—cyclin-dependent kinase inhibitor 1 (CDKN1A); p53—tumor protein 53; PARP—poly(ADP-ribose)-1 polymerase; PI3K—phosphatidylinositol 3-kinase; TNF—Tumor Necrosis Factor; and TOP2A—Topoisomerase II Alpha.
